# Real-World Data on Characteristics and Management of Patients with Actinic Keratosis: A Cancer Center Experience †

**DOI:** 10.3390/medsci14040474

**Published:** 2026-08-12

**Authors:** Flavia Silvestri, Francesca Pepe, Sara Gandini, Aurora Gaeta, Paola Queirolo, Giulio Tosti

**Affiliations:** 1 Dermato-Oncology Unit, European Institute of Oncology, IRCCS, 20141 Milan, Italy; francesca.pepe@ieo.it (F.P.); giulio.tosti@ieo.it (G.T.); 2 Molecular and Pharmaco-Epidemiology Unit, Department of Experimental Oncology, European Institute of Oncology, IRCCS, 20139 Milan, Italy; sara.gandini@ieo.it (S.G.); aurora.gaeta@ieo.it (A.G.); 3 Department of Statistics and Quantitative Methods, University of Milano-Bicocca, 20126 Milan, Italy; 4 Division of Medical Oncology for Melanoma, Sarcoma and Rare Tumors, European Institute of Oncology, IRCCS, 20141 Milan, Italy; paolinaque@gmail.com

**Keywords:** real-world data, dermato-oncology, actinic keratosis

## Abstract

**Background/Objectives**: Actinic keratosis is an intraepithelial lesion that may progress into squamous cell carcinoma; therefore, all lesions should be treated. The study aimed to investigate the potential correlations between patient and lesion characteristics, treatment option choice, and clinical outcomes, analyzing long-term real-world data. **Methods**: A retrospective study was conducted in a specialized hospital in Milan. Data on patients with actinic keratoses were collected from January 2018 to July 2024. **Results**: A total of 369 patients were included, with normal weight (58%), higher educational level (83%), personal skin cancer history (51%), childhood sunburns (68%), and face localization (70%), with multiple contiguous distribution (54%) of lesions. In total, 45.5% received multiple treatments. Field-directed therapies were prescribed in 84% of cases (32% of complete clearance). A total of 43% were lost to follow-up. Higher educational level, personal and familiar skin cancer history, previous atypical naevus excision, higher naevus count, and multiple treatments (*p* < 0.05) were associated with regularity in visit attendance. In multivariable analysis, immunosuppression (OR = 5.01 [0.97, 29.5], *p* = 0.056) and multiple contiguous lesions (OR = 4.62 [1.75, 14.7], *p* = 0.004) were found to be significantly associated with incomplete clinical response. **Conclusions**: Real-world data on patients with actinic keratoses may help identify more personalized management strategies that influence treatment outcomes and adherence to follow-up.

## 1. Introduction

Actinic keratosis (AK) is considered one of the most common dermatologic conditions diagnosed in adult patients, accounting for at least 10% of all dermatological visits [1,2].

A recent meta-analysis reported a global prevalence of 14% and an estimated incidence rate of 1.9 per 1000 person-year for AKs [3]. In Italy, a high prevalence of AK in dermatology outpatients has been reported, involving around 27% of the population ≥ 30 [4].

The major risk factors associated with AKs are cumulative ultraviolet radiation exposure, older age, Fitzpatrick phototype I-II, some genetic disorders, and immunosuppression [4,5,6,7,8].

Furthermore, the associations between other sociodemographic factors, such as gender, body mass index (BMI), and educational level, are still debated [4,5,6,7,8].

The World Health Organization (WHO) defines AK as an intraepithelial neoplastic lesion. However, it considers AK a carcinoma precursor that may progress into invasive cutaneous squamous cell carcinoma (cSCC) [9], with an estimated progression rate of up to 10% [10].

Although AKs can spontaneously regress in some cases, current European Guidelines recommend always treating AKs for their unpredictable clinical behavior [11].

Various effective treatment options do exist for AKs, which can be divided into lesion-directed therapies (LDTs), usually directed at individual lesions, and field-directed therapies (FDTs) for multiple and subclinical lesions.

Although a standard treatment for AKs has not yet been established, FDTs are considered the more appropriate approach since they can be home-administered and target field cancerization as well [11].

The efficacy and tolerability data of the most utilized FDTs are usually retrieved from the results of randomized controlled trials, in which a rigorous selection of patients and short-term follow-up may generate non-representative routine clinical practice samples. Real-world data (RWD) can support clinical trial studies by providing additional information on a more generalizable population and other areas, such as the disease’s natural history, effectiveness studies, and specific patient subgroups (e.g., with multiple comorbidities). Furthermore, utilizing existing data can significantly reduce time and resources for data collection, potentially maximizing cost-effectiveness.

This study aimed to investigate potential correlations between patient characteristics, lesion characteristics, treatment option choice, and related clinical outcomes in patients with AKs. It analyzed real-life data from an internal database of a specialized cancer treatment center during a six-year observation period. Moreover, follow-up data were collected to better comprehend the critical points, such as loss to follow-up, in managing patients with AKs in a real-life setting.

## 2. Materials and Methods

### 2.1. Study Design

This is a retrospective, observational study conducted in a specialized cancer treatment center in Milan, Italy, with a six-year follow-up period.

The study was performed in accordance with the revised Declaration of Helsinki and approved by the local Ethics Committee of the European Institute of Oncology of Milan (UID4262, 20 July 2023).

### 2.2. Study Population

Patients aged ≥18 years with a current clinical diagnosis of AK on all body areas, treated or followed up at the Dermato-oncology Unit of the European Institute of Oncology in Milan between January 2018 and July 2024 were recruited.

All patients gave written informed consent to participate in the study.

Patients with incomplete data and missing written informed consent were excluded from the analysis, resulting in 369 patients.

### 2.3. Data Collection

Patient information was retrieved from an internal database and included demographic data (age, gender, BMI, and educational level); medical history (comorbidities, concomitant medications, personal previous and/or current cancer history, immunosuppression, personal and family history of skin cancer); patient clinical data (Fitzpatrick phototype, chronic sun-damage (CSD) evaluated as the clinical presence of lentigo solaris on sun-exposed areas, and melanocytic naevus count on the forearms); evaluation for the presence of AK risk factor (childhood sunburns and ultraviolet radiation exposure); lesion characteristics (site, distribution, and clinical aspect classified according to the Olsen classification); current and past treatments and their outcomes. The treatment modalities were grouped as follows:

- FDTs (methyl aminolevulinate (MAL) photodynamic therapy (PDT), 3% diclofenac sodium/hyaluronic acid gel, ingenol mebutate, 1% tirbanibulin ointment, 5% 5-fluorouracil (5-FU), imiquimod, 0.5% 5-FU/10% salicylic acid-lacquer);
- LDTs (surgical excision, cryotherapy, curettage and/or electrodesiccation, laser therapy);
- Observation;
- Chemoprevention with nicotinamide.

The clinical response was evaluated once after the end of treatment, according to outpatient-scheduled follow-up visits.

Clinical responses were grouped as follows:

- Complete clearance (CC) is characterized by a 100% reduction in treated AKs’ lesions.
- Partial clearance (PC) is defined by an AKs reduction between 75 and 99% of treated AKs.
- Stable disease (SD) consists of AKs reduction < 75%.
- Development of new lesions after the end of treatment.

### 2.4. Statistical Analysis

Absolute frequencies and percentages were reported to summarize categorical variables. Continuous variables were reported as median and interquartile range (IQR).

A multivariable logistic regression model assessed the association between the available variables and the response (CC, PC, SD, and the development of new lesions). Odds ratios (ORs) were estimated to quantify the strength and direction of these associations, as well as the OR 95% confidence intervals (CIs).

Variables included in the multivariable model were selected based on their significance in the univariate analysis. Statistical significance was set at t *p* < 0.05, and all analyses were performed using R version 4.3.1 (16 June 2023).

Alluvial diagrams are presented to show changes in time in response status before and after treatments.

## 3. Results

### 3.1. Patient Characteristics

Patient characteristics are provided in Table 1. A total of 369 patients with AKs were included, of which 168 (45.5%) were male and 201 (54%) were women, with a mean age at diagnosis of 69 years (IQR: 57–76); see Table 1. Most patients (58%) presented a normal weight, with an overall median BMI of 23.8 (IQR: 21.2, 26.0).

The educational level was high, with 155 (42%) high school graduates and 153 (41%) university graduates. Comorbidities were present in 209 cases (57%), including 77 patients (21%) with non-cutaneous personal cancer history, of which 23 cases (6%) had concomitant or recent cancer history of <5 years. Most patients (58%) used concomitant medications, of which 154 (42%) used <3 medications. Only 10 patients (2.7%) were immunocompromised. The most represented Fitzpatrick phototypes were types II (65%) and III (30%). One hundred and eighty-two patients (49%) had no previous skin cancers, while 187 (51%) patients reported a positive skin cancer history with 12 (6.5%) melanoma cases, 35 (51%) NMSC cases, and both skin tumors in 14 cases (20.8%). Familiar skin cancer history was positive in only 68 (18%) of patients, with the highest rate for NMSCs reported in 35 cases (51%), followed by melanoma (19 cases) and combined skin tumors (14 cases). Clinically, most of the patients (95%) had no history of histopathologically diagnosed atypical melanocytic naevi.

Sunburns during childhood and lifetime sun exposure were reported in 252 cases (68%) and 321 (87%) cases, respectively. Of note, 265 patients (71.8%) reported outdoor sun exposure as the most frequent modality, with only 56 patients (15%) reporting indoor sun exposure. In 48 patients (13%), the data were not specified. Lentigo solaris in sun-exposed areas was present in 91 patients (25%). Most of the patients (87%) showed less than 20 melanocytic naevus counts on the forearms.

### 3.2. Lesion Characteristics

Most of the AK localizations were limited to the face (70%), followed by face and scalp (8.4%), limited to the scalp (8.2%), face and rest of the body (6.5%), rest of the body (3.3%), face and scalp and rest of the body (2.7%), and scalp and rest of the body (0.5%).

Of note, 198 (54%) patients showed a multiple contiguous distribution of the lesions, followed by isolated lesions in 35% of cases, while only 41 patients (11%) showed a multiple non-contiguous lesion distribution. In one case of multiple lesions, the distribution pattern was not specified.

Clinically, when specified, the AKs were classified as Olsen grade 1 in 32 cases (37%) and grade > 1 in 36 cases (41%). Nineteen (22%) AKs were pigmented.

All the lesion characteristics details are provided in Table 2.

### 3.3. Treatment and Outcomes

More than half of patients (51.5%) had no previous AK treatments, while 178 (48.2%) received previous treatments, of which the most frequent was the combined approach with both FDTs and LDTs, used in 81 (45.5%) of cases, followed by FDTs (41.6%) and LDTs (13%).

Among previously treated patients, 94 (52.8%) had PC, 55 (31%) had SD, and 25 (14%) had CC.

Only four cases (2.2%) developed new lesions after the end of treatment.

Interestingly, FDTs were the most frequently prescribed treatment (84%), followed by observation (11.6%), LDTs (9.8%), and combined approaches (6.1%).

Only 10% of patients received oral chemoprevention with nicotinamide.

Regarding the treatment outcome, 118 patients (32%) had CC of the AKs, while 18 patients (4.9%) and 15 patients (4.1%) experienced PC and SD, respectively. Only one patient developed new lesions at the end of treatment.

Interestingly, 160 patients (43%) were considered lost to follow-up since they did not attend any follow-up visits, while 57 patients continued treatment by the end of the study.

Out of 369 patients, 209 patients regularly attended follow-up visits.

We found that regularity in follow-up attendance was more frequent among patients with higher educational level, personal and familiar skin cancer history, previous atypical naevus excision, higher naevus count (>20 on the forearms), and multiple prescribed treatments (*p* < 0.05) (Table 3).

Overall, the median number of encounters for AKs was 2.0 (IQR: 1.0, 5.0).

**Figure 1:**
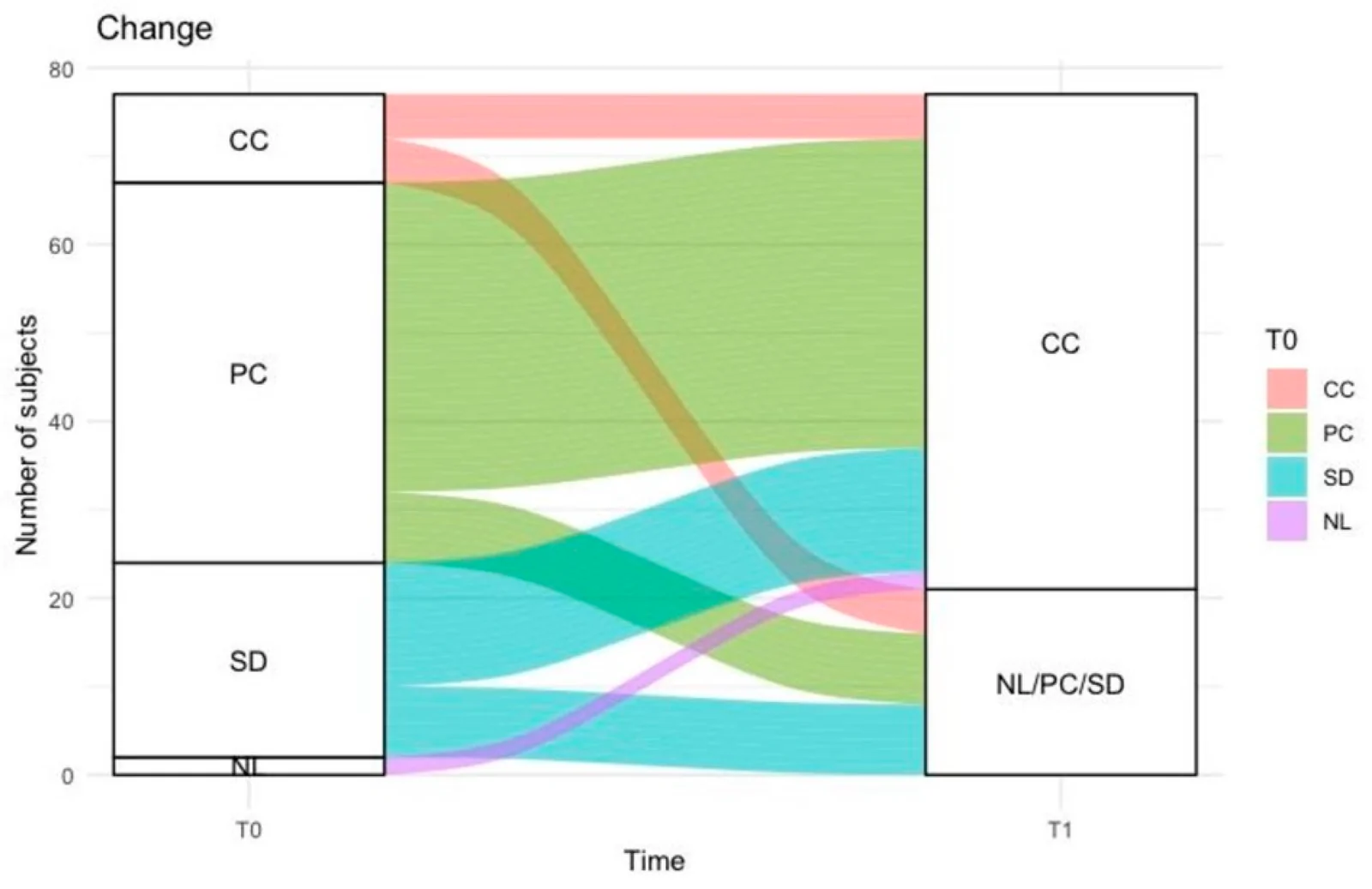
Flow diagram comparing the treatment response between patients previously and currently treated for AKs. A high proportion of previously treated patients who have not achieved CC, including PC, SD, and the development of new lesions, obtained CC after current treatments.

All the treatment and outcome details are provided in Table 2 and Table 3 and Figure 1.

In addition, a consistent proportion of previously treated patients maintained CC after current treatments.

### 3.4. Multivariable Analysis

In multivariable analysis (Table 4), immunosuppression (OR = 5.01 [0.97, 29.5] *p* = 0.056) and multiple contiguous lesions (OR = 4.62 [1.75, 14.7] *p* = 0.004) were found to be significantly more associated with incomplete clinical response (PC, SD, and developing new lesions).

Other patient and lesion characteristics were not found to be significantly associated with response.

## 4. Discussion

AKs are common skin lesions that can occasionally transform into cSCC. However, they are usually harmless, so they are largely managed in dermatology outpatients, resulting in widely incomplete epidemiological and clinical data on AKs.

Nowadays, various treatment options for AKs do exist. Several clinical trials have investigated the efficacy and tolerability of different types of treatments; nevertheless, it is important to emphasize that data provided by clinical trials are often non-representative of daily clinical practice, frequently not considering the importance of a personalized treatment choice. Therefore, RWD on particular subgroups of AK patients is limited.

This hospital-based cohort study aimed to investigate the overall management of patients with AK, analyzing RWD on patient and lesion characteristics, treatment outcome, and follow-up data in a skin cancer specialized center.

Over time, various studies have investigated the major risk factors associated with AKs, such as age, skin phototype, lifetime sun exposure, and immune system status [4,5,6,7,8,11,12].

Many studies reported male gender, especially bald males, to be associated with an increased risk of severe actinic skin damage, with a consequent higher prevalence of AK [4,7,8].

Conversely, in our study, female gender was predominant among AK patients (54%).

One reason for the prevalence of women in our population might be that the center is highly specialized in breast cancer treatment.

Similar to other studies [4,5,6,7,8], age > 50 years (mean 69 years), Fitzpatrick II skin type, and higher educational levels were the most frequently observed characteristics in our patients.

In a case–control study, Traianou et al. reported a higher risk for AKs with thiazide diuretics and cardiac drug intake [5]. These data are consistent with our results since most of our patients presented comorbidities, in particular cardiovascular diseases, such as hypertension and hypercholesterolemia, usually treated with more than three medications.

Despite our study being hospital-based cancer treatment, personal cancer history was not significantly associated with AK incidence in our cohort. This fact may be partly explained by a pre-existing awareness of the premalignant nature of AKs that may progress into cSCC by patients accessing our Dermatoncology unit.

Half of the patients had a positive skin cancer history, of which 30% reported previous Nonmelanoma skin cancer (NMSC). This is partly because AK is considered a potentially incipient cSCC, which shares with cSCC the same high cumulative UV exposure pathogenesis. This fact is further confirmed by the higher percentage of childhood sunburns and lifetime UV-exposure behavior in large population-based studies of people at risk of developing skin cancers [4,5,6,7,8].

Our results are consistent with the literature, showing patients reporting childhood sunburns (68%) and outdoor sun exposure (71.8%). Only a few patients reported indoor sun exposure, possibly as a result of broad prevention campaigns against skin cancer.

As lesions arising in chronically sun-exposed areas, AKs are commonly localized in the head and neck region. In our study, the most frequent localization was the face (70%), with a predominant multiple contiguous lesion distribution (54%), which is consistent with the concept of FC. This highlights that the skin surrounding AKs presents an increased risk of progression [4,5,6,7,8].

According to the current European Guidelines [11], most of our patients (84%) received FDT prescriptions, which emphasize FDTs as the preferable treatment option.

Although there is no standard treatment for AKs, and not all treatment options are suitable for all patients, FDTs are the only ones able to target subclinical lesions and have the advantage of being home-administered [7]. Moreover, novel FDT options are emerging in the AK landscape, such as Tirbanibulin, a topical microtubule inhibitor with only a 5-day treatment duration for face and scalp AKs [13].

Given its recent approval in Europe (July 2021), only a few cases have been treated with Tirbanibulin. However, numerous studies have proven it to be an effective and safe FDT option [13,14,15].

About half of our patients received at least one previous treatment for AK, including LDTs and FDTs.

Outcome data were relatively limited due to some patients still receiving treatment by the end of the observation period, but mainly due to the elevated number of LTF patients (43%).

This high percentage could be explained by either a lack of patient awareness, which downplays the importance of close monitoring in AK management, or long waiting times for hospital-based appointments, leading patients to make appointments in other dermatology outpatients.

As for predictive factors of AK treatment response, a consensus paper analyzed different clinical scenarios and populations, reporting that therapy should be personalized based on previous treatments, patient, and lesion characteristics [16].

Our study found a multivariable association between immune system status and lesion distribution with no CC rates after treatment.

Notably, immunosuppression (OR = 5.01 [0.97, 29.5] *p* = 0.056) and multiple contiguous lesion distribution (OR = 4.62 [1.75, 14.7] *p* = 0.004) were significantly associated with no CC after treatment, including PC, SD, and new lesion development.

Immunosuppression is a well-known risk factor for the development of malignancies, as well as for AKs, especially for solid organ transplant recipients compared to the overall population, with 80% who develop AKs during their lifetime and up to 30% of them suffering from more than five lesions [12].

This subgroup of patients frequently shows treatment resistance and multiple lesions over large FC; thus, authors recommend management in specialized centers [11]. Moreover, secondary prevention by switching to mTOR inhibitors and prescribing photoprotection and chemoprevention with retinoids is also highly recommended [11,12].

In our study, multiple contiguous lesion distribution could be correlated with reduced CC since it represented the most frequent distribution. Most importantly, it involves a wider treatment area, including clinical and subclinical lesions, which could appear misleading about the actual AKs’ depth of extension and severity.

As there is no standardized approach to defining FC and reproducible clinical and histological grading systems for assessing the extent of AKs, emerging non-invasive diagnostic techniques could provide a more accurate AK severity evaluation that could potentially correlate with its risk of malignancy [12,17].

Since AKs frequently affect cosmetically critical body areas, a cost-effective therapy with good cosmetic results is mandatory for the best treatment choice.

Margit et al. reported real-life clinical practice data on patients with AK, showing higher lesion severity and much higher costs of FDTs in real-life settings than in clinical trials [18].

In our study, the median number of encounters for AKs was relatively low, 2.0 (IQR: 1.0, 5.0), largely due to the elevated percentage of LTF patients, but we expected much higher numbers in terms of encounters and costs compared to our results.

Regular follow-up visit attendance was significantly associated with higher educational level, personal and familiar history of skin cancer, previous melanocytic naevus excision, melanocytic nevus count >20 on the forearms, and FDT prescription.

All these factors could be associated with an increased awareness of the importance of skin cancer prevention and early detection. Furthermore, health services could be more easily accessible and available for patients with higher educational levels.

On the other hand, the high number of LTFs could be explained by the overall benign behavior of AKs and the effectiveness of the home-administered treatments, which allow patients not to attend follow-up visits.

Conversely, the different treatment duration and tolerability, especially for FDTs, could affect treatment adherence and effectiveness, leading to a higher proportion of patients attending close monitoring visits.

The main limitation of this study is its retrospective design, which utilized already existing data, which could be missing or incomplete. Some relevant information about treatment tolerability and potential progression into cSCC was not retrieved in this case. However, information biases have been minimized by the large population-based cohort investigated. Furthermore, the findings of our study may form the basis on which new prospective studies are planned.

## 5. Conclusions

The management of AKs is still challenging, especially for specific clinical scenarios and subgroups of patients. Our large population-based cohort study provided RWD of a cancer treatment hospital on patient and lesion characteristics, which may influence treatment outcomes and patient adherence to follow-up.

Immunosuppression and multiple contiguous lesion distribution were significantly associated with no CC after treatment. Moreover, higher educational level, personal and familiar history of skin cancer, previous melanocytic naevus excision, overall nevus count > 20, and FDT prescription were significantly associated with regular follow-up visit attendance.

Further studies in the real-world setting are needed to provide more accurate data on the effectiveness, tolerability, and treatment adherence of home-administered treatments. Emerging noninvasive diagnostic techniques could improve AK extension depth evaluation to identify a more personalized treatment selection.

## Figures and Tables

**Table 1:** Patient characteristics.

Characteristics	N = 369 ^1^
**Mean Age at first diagnosis (IQR)**	69 (57, 76)
**Gender**	
Male	168 (45.5%)
Female	201 (54%)
**Median BMI (IQR)**	23.8 (21.2, 26.0)
Underweight	20 (5.4%)
Normal weight	213 (58%)
Overweight	115 (31%)
Obesity	21 (5.7%)
**Education**	
Elementary school	61 (17%)
High school	155 (42%)
University	153 (41%)
**Comorbidities**	
No	160 (43%)
Yes	209 (57%)
**Concomitant medications**	
No	156 (42%)
≤3 medications	154 (42%)
>3 medications	59 (16%)
**Personal cancer history**	
No	292 (79%)
≤5 years/concomitant	23 (6%)
>5 years/indefinite	54 (15%)
**Immunosuppression**	
No	359 (97%)
Yes	10 (2.7%)
**Fitzpatrick phototype**	
I	13 (3.5%)
II	241 (65%)
III	111 (30%)
IV	4 (1.1)
**Personal skin cancer history**	
No	182 (49%)
Melanoma	12 (6.5%)
NMSC	55 (30%)
Multiple skin tumors	120 (32.5%)
**Family skin cancer history**	
No	301 (82%)
Melanoma	19 (28%)
NMSC	35 (51%)
Multiple skin tumors	14 (20.8%)
**Previous melanocytic naevi excision**	
No	350 (95%)
Yes	19 (5.1%)
**Childhood sunburns**	
No	117 (32%)
Yes	252 (68%)
**Sun exposure**	
Indoor	56 (15%)
Outdoor	265 (71.8%)
Not specified	48 (13%)
**Lentigo solaris (CSD)**	
No	278 (75%)
Yes	91 (25%)
**Melanocytic naevus count**	
<20 on the forearms	322 (87%)
>20 on the forearms	47 (13%)

^1^ Median (Q1, Q3); n (%). BMI (body mass index); CSD (chronic sun damage); IQR (interquartile range); NMSC (nonmelanoma skin cancer).

**Table 2:** Lesion characteristics.

Characteristics	N = 369 ^1^
**Site**	
Face	259 (70%)
Face and rest of the body	24 (6.5%)
Scalp	30 (8.2%)
Scalp and rest of the body	2 (0.5%)
Face and scalp	31 (8.4%)
Face and scalp and rest of the body	10 (2.7%)
Rest of the body	12 (3.3%)
Unknown	1 (0.3%)
**Distribution**	
Isolated lesion	129 (35%)
Multiple contiguous lesions	198 (54%)
Multiple non- contiguous lesions	41 (11%)
Multiple not specified	1 (0.3%)
**Clinical aspect**	
Grade 1	32 (37%)
Grade > 1	36 (41%)
Pigmented	19 (22%)
Not specified	282 (76.4%)
**Previous treatments**	
None	190 (51.5%)
FDTs	74 (41.6%)
LDTs	23 (13%)
Multiple treatments	81 (45.5%)
Unknown	1 (0.3%)
**Previous treatments outcome**	
CC	25 (14%)
PC	94 (52.8%)
SD	55 (31%)
New lesions	4 (2.2%)
**Treatment**	
None	43 (11.6%)
FDTs	274 (84%)
LDTs	32 (9.8%)
Multiple treatments	20 (6.1%)
**Nicotinamide.**	
No	333 (90%)
Yes	36 (9.8%)
**Treatment outcome**	
**CC**	**118 (32%)**
PC	18 (4.9%)
SD	15 (4.1%)
New lesions	1 (0.3%)
Ongoing	57 (15%)
Lost to follow-up	160 (43%)
**Median number of encounters for AKs (IQR)**	1.0 (1.0, 5.0)

^1^ Median (Q1, Q3); n (%). AK (actinic keratosis); CC (complete clearance); FDTs (field-directed therapies); IQR (interquartile range); LDTs (lesion-directed therapies); PC (partial clearance); SD (stable disease).

**Table 3:** Patient characteristics associated with regularity in follow-up visit attendance.

Characteristics	Regular in Follow-Up	Lost to Follow-Up	*p*-Value ^2^
	N = 209 ^1^	N = 160 ^1^	
Education			0.007
Elementary school	24 (11%)	37 (23%)	
High school	89 (43%)	66 (41%)	
University	96 (46%)	57 (36%)	
Personal skin cancer history	129 (62%)	74 (46%)	0.003
Familiar skin cancer history	46 (22%)	22 (14%)	0.043
Previous melanocytic naevus	15 (7.2%)	4 (2.5%)	0.044
excision			
Melanocytic naevus count	33 (16%)	14 (8.8%)	0.044
(>20 on the forearms)			
Treatment prescriptions			0.008
FDTs	166 (81%)	108 (90%)	
LDTs	21 (10%)	11 (9.2%)	
Multiple treatments	19 (9.2%)	1 (0.8%)	

^1^ n (%), ^2^ Pearson’s Chi-squared test; Fisher’s exact test. FDTs (field-directed therapies); LDTs (lesion-directed therapies).

**Table 4:** Multivariable association with no complete clearance after treatment.

Characteristic	OR ^1^	95% CI ^1^	*p*-Value
Immunosuppression			
No	-	-	
Yes	5.01	0.97, 29.5	0.056
Lesion distribution			
Isolated	-	-	
Multiple contiguous	4.62	1.75, 14.7	0.004
lesions			
Multiple non-	2.78	0.51, 13.3	0.2
contiguous lesions			

^1^ OR = Odds Ratio, CI = Confidence Interval.

## Data Availability

The data presented in this study are available on request from the corresponding author due to privacy and ethical reasons.

## Abbreviations

The following abbreviations are used in this manuscript:

AK	Actinic keratosis
BMI	Body mass index
WHO	World Health Organization
cSCC	Cutaneous squamous cell carcinoma
LDTs	Lesion-directed therapies
FDTs	Field-directed therapies
RWD	Real-world data
CSD	chronic sun-damage
MAL	methyl aminolevulinate
PDT	photodynamic therapy
5-FU	5% 5-fluorouracil
CC	Complete clearance
PC	Partial clearance
SD	Stable disease
OR	Odds ratio
CI	confidence intervals
IQR	interquartile range
NMSC	Nonmelanoma skin cancer

## References

[1] Noels E.C., Hollestein L.M., Egmond S., Lugtenberg M., Nistelrooij L.P.J., Bindels P.J.E., Lei J., Stern R.S., Nijsten T., Wakkee M. (2019). Healthcare utilization and management of actinic keratosis in primary and secondary care: A complementary database analysis. Br. J. Dermatol..

[2] Berman B., Armstrong A., Lebwohl M., Grada A., Bhatia N., Patel V.A., Rigel D., Del Rosso J., Schlesinger T., Kircik L. (2023). Developing a questionnaire for assessing clinician- and patient-reported outcomes in actinic keratosis: Results from an expert panel. JAAD Int..

[3] George C.D., Lee T., Hollestein L.M., Asgari M.M., Nijsten T. (2024). Global epidemiology of actinic keratosis in the general population: A systematic review and meta-analysis. Br. J. Dermatol..

[4] Fargnoli M.C., Altomare G., Benati E., Borgia F., Broganelli P., Carbone A., Chimenti S., Donato S., Girolomoni G., Micali G. (2017). Prevalence and risk factors of actinic keratosis in patients attending Italian dermatology clinics. Eur. J. Dermatol..

[5] Traianou A., Ulrich M., Apalla Z., De Vries E., Bakirtzi K., Kalabalikis D., Ferrandiz L., Ruiz-de-Casas A., Moreno-Ramirez D., Sotiriadis D. (2012). Risk factors for actinic keratosis in eight European centres: A case-control study. Br. J. Dermatol..

[6] de Vries E., Trakatelli M., Kalabalikis D., Ferrandiz L., Ruiz-de-Casas A., Moreno-Ramirez D., Sotiriadis D., Ioannides D., Aquilina S., Apap C. (2012). Known and potential new risk factors for skin cancer in European populations: A multicentre case-control study. Br. J. Dermatol..

[7] Li Y., Wang J., Xiao W., Liu J., Zha X. (2022). Risk Factors for Actinic Keratoses: A Systematic Review and Meta-Analysis. Indian J. Dermatol..

[8] Schaefer I., Augustin M., Spehr C., Reusch M., Kornek T. (2014). Prevalence and risk factors of actinic keratoses in Germany--analysis of multisource data. J. Eur. Acad. Dermatol. Venereol..

[9] Elder D.E., Massi D., Scolyer R.A., Willemze R. (2018). WHO Classification of Skin Tumours.

[10] Fuchs A., Marmur E. (2007). The kinetics of skin cancer: Progression of actinic keratosis to squamous cell carcinoma. Dermatol. Surg..

[11] Kandolf L., Peris K., Malvehy J., Mosterd K., Heppt M.V., Fargnoli M.C., Berking C., Arenberger P., Bylaite-Bučinskiene M., Del Marmol V. (2024). European consensus-based interdisciplinary guideline for diagnosis, treatment and prevention of actinic keratoses, epithelial UV-induced dysplasia and field cancerization on behalf of European Association of Dermato-Oncology, European Dermatology Forum, European Academy of Dermatology and Venereology and Union of Medical Specialists (Union Européenne des Médecins Spécialistes). J. Eur. Acad. Dermatol. Venereol..

[12] Thamm J.R., Schuh S., Welzel J. (2024). Epidemiology and Risk Factors of Actinic Keratosis. What is New for The Management for Sun-Damaged Skin. Dermatol. Pract. Concept..

[13] Blauvelt A., Kempers S., Lain E., Schlesinger T., Tyring S., Forman S., Ablon G., Martin G., Wang H., Cutler D.L. (2021). Phase 3 Trials of Tirbanibulin Ointment for Actinic Keratosis. N. Engl. J. Med..

[14] Pellacani G., Schlesinger T., Bhatia N., Berman B., Lebwohl M., Cohen J.L., Patel G.K., Kunstfeld R., Hadshiew I., Lear J.T. (2024). Efficacy and safety of tirbanibulin 1% ointment in actinic keratoses: Data from two phase-III trials and the real-life clinical practice presented at the European Academy of Dermatology and Venereology Congress 2022. J. Eur. Acad. Dermatol. Venereol..

[15] Kirchberger M.C., Gfesser M., Erdmann M., Schliep S., Berking C., Heppt M.V. (2023). Tirbanibulin 1% Ointment Significantly Reduces the Actinic Keratosis Area and Severity Index in Patients with Actinic Keratosis: Results from a Real-World Study. J. Clin. Med..

[16] Moscarella E., Di Brizzi E.V., Casari A., De Giorgi V., Di Meo N., Fargnoli M.C., Lacarrubba F., Micali G., Pellacani G., Peris K. (2020). Italian expert consensus paper on the management of patients with actinic keratoses. Dermatol. Ther..

[17] Lyle R.E., Tran L.H., Eisen D.B. (2025). Innovations in Actinic Keratosis. Dermatol. Clin..

[18] Van Rijsingen M.C., Seubring I., Grutters J.P., Maessen-Visch M.B., Alkemade H.A., Van Doorn R., Groenewoud H., van de Kerkhof P.C., Van Der Wilt G.J., Gerritsen M.J.P. (2016). Real-life Data on Patient Characteristics, Cost and Effectiveness of Field-directed Treatment for Actinic Keratoses: An Observational Study. Acta Derm. Venereol..

